# Mitochondrial aminoacyl-tRNA synthetases trigger unique compensatory mechanisms in neurons

**DOI:** 10.1093/hmg/ddad196

**Published:** 2023-11-17

**Authors:** Oliver Podmanicky, Fei Gao, Benjamin Munro, Matthew J Jennings, Veronika Boczonadi, Denisa Hathazi, Juliane S Mueller, Rita Horvath

**Affiliations:** Department of Clinical Neurosciences, John Van Geest Centre for Brain Repair, University of Cambridge, Ed Adrian Building, Robinson Way, Cambridge, CB2 0PY, United Kingdom; Department of Clinical Neurosciences, John Van Geest Centre for Brain Repair, University of Cambridge, Ed Adrian Building, Robinson Way, Cambridge, CB2 0PY, United Kingdom; Department of Clinical Neurosciences, John Van Geest Centre for Brain Repair, University of Cambridge, Ed Adrian Building, Robinson Way, Cambridge, CB2 0PY, United Kingdom; Department of Clinical Neurosciences, John Van Geest Centre for Brain Repair, University of Cambridge, Ed Adrian Building, Robinson Way, Cambridge, CB2 0PY, United Kingdom; Department of Neurology, Columbia University, 630 West 168^th^ St, New York, NY 10032, United States; Biosciences Institute, International Centre for Life, Faculty of Medical Sciences, Newcastle University, Central Parkway, Newcastle upon Tyne, NE1 3BZ, United Kingdom; Department of Clinical Neurosciences, John Van Geest Centre for Brain Repair, University of Cambridge, Ed Adrian Building, Robinson Way, Cambridge, CB2 0PY, United Kingdom; Department of Clinical Neurosciences, John Van Geest Centre for Brain Repair, University of Cambridge, Ed Adrian Building, Robinson Way, Cambridge, CB2 0PY, United Kingdom; Dubowitz Neuromuscular Centre, Department of Neuropathology, Institute of Neurology, Queen Square, London, WC1N 3BG, United Kingdom; Department of Clinical Neurosciences, John Van Geest Centre for Brain Repair, University of Cambridge, Ed Adrian Building, Robinson Way, Cambridge, CB2 0PY, United Kingdom

**Keywords:** aminoacyl-tRNA synthetase, neurological disease, protein synthesis, mitochondrial biology

## Abstract

Mitochondrial aminoacyl-tRNA synthetase (mt-ARS) mutations cause severe, progressive, and often lethal diseases with highly heterogeneous and tissue-specific clinical manifestations. This study investigates the molecular mechanisms triggered by three different mt-ARS defects caused by biallelic mutations in *AARS2, EARS2*, and *RARS2*, using an in vitro model of human neuronal cells. We report distinct molecular mechanisms of mitochondrial dysfunction among the mt-ARS defects studied. Our findings highlight the ability of proliferating neuronal progenitor cells (iNPCs) to compensate for mitochondrial translation defects and maintain balanced levels of oxidative phosphorylation (OXPHOS) components, which becomes more challenging in mature neurons. Mutant iNPCs exhibit unique compensatory mechanisms, involving specific branches of the integrated stress response, which may be gene-specific or related to the severity of the mitochondrial translation defect. RNA sequencing revealed distinct transcriptomic profiles showing dysregulation of neuronal differentiation and protein translation. This study provides valuable insights into the tissue-specific compensatory mechanisms potentially underlying the phenotypes of patients with mt-ARS defects. Our novel in vitro model may more accurately represent the neurological presentation of patients and offer an improved platform for future investigations and therapeutic development.

## Introduction

Healthy mitochondria are essential for maintaining metabolic homeostasis and ATP production via oxidative phosphorylation (OXPHOS). Disruption to mitochondrial biogenesis or energy production underlies a broad range of metabolic diseases, primarily affecting skeletal muscle and the nervous system. Mitochondrial diseases are often progressive and fatal, representing the most common group of inherited metabolic disorders with no effective therapy [1, 2]. Mitochondrial disorders are caused by mutations in either the 16.5 kb mitochondrial DNA (mtDNA) encoding 13 OXPHOS proteins, or in over 300 nuclear-encoded mitochondrial proteins. Mitochondrial protein translation requires a separate apparatus for synthesizing mtDNA-encoded proteins, separate from eukaryotic (cytosolic) translation. Aminoacyl tRNA synthetase (ARS) enzymes catalyze the charging of amino acids to their cognate tRNA molecules to facilitate their delivery to the ribosome or mitoribosome, the site of protein translation. There are 17 mt-ARS enzymes, which are present in all cells, as they charge mt-tRNAs with the corresponding (cognate) amino acid to enable synthesis of the 13 mtDNA-encoded proteins [1, 2]. They are distinct from the 18 cytosolic ARS, which aminoacylate tRNAs in the cytosol. Two ARS are present both in the cytosol and mitochondria (glycyl-tRNA synthetase (*GARS1)* & lysyl-tRNA synthetase *(KARS1)*). Mutations in both mitochondrial and cytosolic ARS cause tissue-specific phenotypes frequently involving neurodegeneration [3]. Mutant mt-ARS may cause abnormal amino acid composition of mtDNA-encoded proteins, leading to combined respiratory chain deficiency [1, 2].

Originally thought to be ultra-rare, mt-ARS mutations are major causes of mitochondrial diseases in children [3, 4]. Paradoxically, all mt-ARS are present in each cell, but their mutations affect different cells and organs, most frequently the brain, leading to leukoencephalopathy with thalamus and brainstem involvement and high level of lactate (mitochondrial glutamyl-tRNA synthetase, *EARS2*), leukoencephalopathy with brain stem and spinal cord involvement and lactate elevation (mitochondrial aspartyl-tRNA synthetase, *DARS2)*, leukoencephalopathy with ovarian failure (mitochondrial alanyl-tRNA synthetase, *AARS2*), pontocerebellar hypoplasia (mitochondrial arginyl-tRNA synthetase, *RARS2*) or epileptic encephalopathy (mitochondrial phenylalanyl-tRNA synthetase, *FARS2*, and mitochondrial cysteinyl-tRNA synthetase, *CARS2*). Different mutations in the same mt-ARS may also lead to diverse clinical presentations. For example, point mutations affecting the editing domain of AARS2 were linked to infantile onset cardiomyopathy [5, 6], whereas mutations targeting its aminoacylation domain have been shown to cause leukoencephalopathy and ovarian failure in women [7]. While some studies have investigated different variants of mt-ARSs in yeast models, there is a lack of physiological human models in the study of mt-ARSs in the context of specific clinical representations [8] and ultimately the underlying tissue-specific mechanisms of different mt-ARS are not understood. Mt-ARS mutations frequently affect tissues with high metabolic demand like the brain and muscle, suggestive of altered bioenergetics due to OXPHOS defect as the primary cause of tissue vulnerability [9]. However, many organs with relatively low energy demands, such as ovaries [10], are also selectively affected by specific mt-ARS mutations, thus OXPHOS dysfunction is not the sole mechanism underlying tissue specificity in ARS disorders [7, 11].

Tissue specificity may rely on nuclear-mitochondrial crosstalk to harmonize protein synthesis in response to the energy demands of the cell. Recently, the integrated stress response (ISR) has emerged as an important pathway investigated for its role in communication between mitochondria and the nucleus [12]. This conserved adaptive pathway has been implicated to slow down cytosolic protein translation and restore cellular homeostasis in response to diverse stimuli, such as mitochondrial stress [13]. The detailed mechanisms of the ISR pathway in different tissues remain unclear, however, prolonged activation of this pathway has been associated with various neurodegenerative diseases and may be harmful [12, 14]. It has been also reported in the heart-specific *Dars2* knock-out mice, suggesting that ISR may contribute to tissue specific manifestations in mt-ARS-related disease [15]. In vivo full body mt-ARS knockout mouse models are usually not viable and tissue-specific knock-out or knock-in models do not fully represent the clinical phenotype observed in patients, highlighting the need for better models [15].

In this study, we characterized cellular mechanisms associated with tissue-specific manifestations in neurons in three different mt-ARS-related disease genes *(EARS2, AARS2, RARS2)* using patient-derived in vitro neurons (see Table 1).

**Table 1:** Overview of the mutations and clinical symptoms of patients used in this study.

Gene	Mutation	Amino acid change	Effect of mutation	Clinical signs	Age	Sex
*AARS2*	c.1709delG c.1188G>A	p. G570Afs*21 splice site	frameshift and premature termination splicing variant resulting in exclusion of exon 8	rapidly progressing leukoencephalopathy, cognitive decline, upper limb tremor, hyperphagia	27 years	M
*EARS2*	c.322C>T c.334G>C	p.R108W p.A112P	both are missense mutations in catalytic domain likely impair aminoacylation activity	childhood-onset ataxia, epilepsy, and leukoencephalopathy with thalamic and brain stem involvement (LTBL)	18 months	F
*RARS2*	c.392T>G c.392T>G	p.F131C p.F131C	missense mutation in catalytic domain likely impairs aminoacylation activity	severe encephalopathy, intractable seizures, and profound developmental delay	2 months	F

## Results

### *AARS2* mutations impair mitochondrial translation in neurons

We developed in vitro induced neuronal progenitor cells (iNPCs) from human primary fibroblasts from patients carrying rare pathogenic variants in the mitochondrial alanyl-tRNA synthetase (*AARS2*), mitochondrial glutamyl-tRNA synthetase (*EARS2)* and mitochondrial arginyl-tRNA synthetase (*RARS2*) genes that result in distinct neurological phenotypes. The patient carrying compound heterozygous *AARS2* mutations (c.1709delG, p.G570Afs*21; c.1188G>A; splicing variant resulting in exclusion of exon 8) presented with rapidly progressing leukoencephalopathy leading to cognitive decline, upper limb tremor and hyperphagia [16, 17]. The patient carrying compound heterozygous *EARS2* mutations (c.322C>T, p.R108W; c.334G>C, p.A112P) had childhood-onset ataxia, epilepsy, and leukoencephalopathy with thalamic and brain stem involvement (LTBL). The patient homozygous for the *RARS2* variant (c.392T>G, p.F131C) presented with severe encephalopathy, intractable seizures, and profound developmental delay [18]. Fibroblasts from these patients and healthy controls were reprogrammed to iNPCs and further differentiated into neurons to investigate the impact of specific mt-ARS variants on mitochondrial dysfunction in neurons.

**Figure 1:**
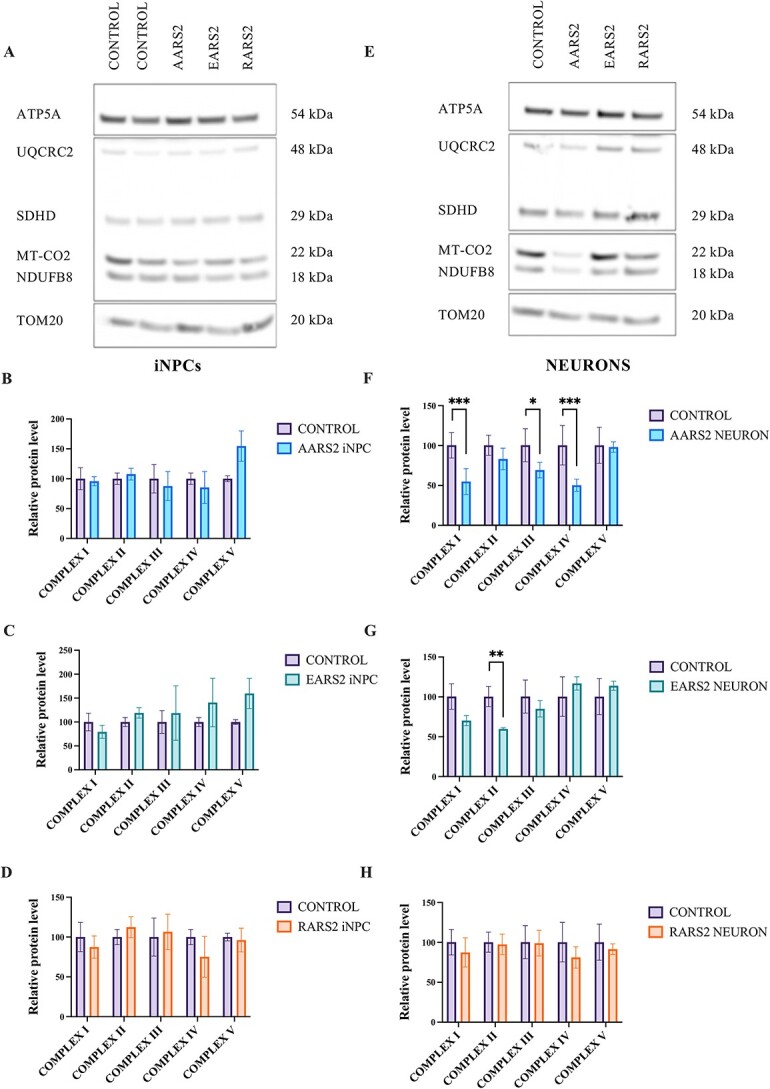
Protein abundance of OXPHOS components in mt-ARS mutant iNPCs and iNPC-derived neurons. Western blot images of OXPHOS subunits and TOM20 in iNPCs (A), and iNPC-derived neurons (E). Quantification of the relative signal intensities normalized to controls and to TOM20 levels as a loading control in complex I (NDUFB8), complex II (SDHD), complex III (UQCRC2), complex IV (MT-CO2), complex V (ATP5A) of iNPCs (B-D) and neurons (F-H). Data shown as mean ± SEM from at least 3 independent experiments, ^*^P ≤ 0.05, ^*^^*^P ≤ 0.01, ^*^^*^^*^P ≤ 0.001, ^*^^*^^*^^*^P ≤ 0.0001, two-way ANOVA (Bonferroni).

To assess the impact of mt-ARS defects on mitochondrial protein synthesis, we used immunoblotting to measure the relative protein levels of individual subunits of OXPHOS complexes I-V in iNPCs and iNPC-derived neurons. Surprisingly, the levels of mtDNA encoded subunits were not significantly altered in any of the mutant iNPC lines compared to controls (Fig. 1A–D). Also, no clear defect of mitochondrial protein synthesis was present in *EARS2* and *RARS2* mutant neurons, suggesting that the defect of mitochondrial translation in these models was prevented by compensatory mechanisms. However, we detected a significant reduction of subunits for complex I (NDUFB8), complex III (UQCRC2), and complex IV (MT-CO2) in *AARS2* mutant neurons (Fig. 1E and F).

**Figure 2:**
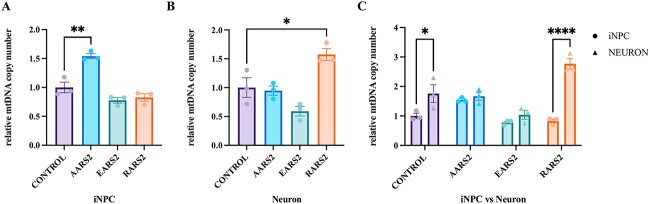
Impact of mt-ARS mutation on mtDNA copy numbers. Relative mtDNA copy numbers in mt-ARS iNPCs vs control (A), and in neurons vs control (B). Data normalized to control mtDNA levels. (C) mtDNA copy numbers during differentiation. Data normalized to control iNPC level. The relative amounts of mtDNA were calculated as mean ΔCt values of the difference in cycle threshold (Ct) of the mitochondrial encoded gene ND1 minus the Ct of the nuclear gene B2M. Data shown as mean ± SEM of 3 independent experiments, ^*^P ≤ 0.05, ^*^^*^P ≤ 0.01, ^*^^*^^*^P ≤ 0.001, ^*^^*^^*^^*^P ≤ 0.0001, one-way ANOVA (Bonferroni).

It has been well-observed that cells carrying pathogenic mtDNA mutations may compensate for impaired protein function by increasing mtDNA replication [19], thus leading us to investigate whether mtDNA copy number was altered in iNPC and iNPC-derived differentiated neurons and if this may underlie the resilience of mt-ARS mutant OXPHOS subunit synthesis seen in Fig. 1. In iNPCs, *AARS2* mutant cells showed elevated mtDNA copy number, while *EARS2* and *RARS2* mutant iNPCs were similar to controls (Fig. 2A). Following differentiation, however, only *RARS2* mutant neurons showed elevated mtDNA copy number (Fig. 2B). Neuronal differentiation requires increasing energy demand, as illustrated in higher mtDNA copy numbers in control and *RARS2* mutant neurons compared to their respective iNPCs. In *AARS2* and *EARS2* mutant neurons mtDNA copy numbers did not increase during differentiation, reflecting impaired compensatory mechanisms (Fig. 2C).

### Mitochondrial respiration is impaired in mutant mt-ARS iNPCs

**Figure 3:**
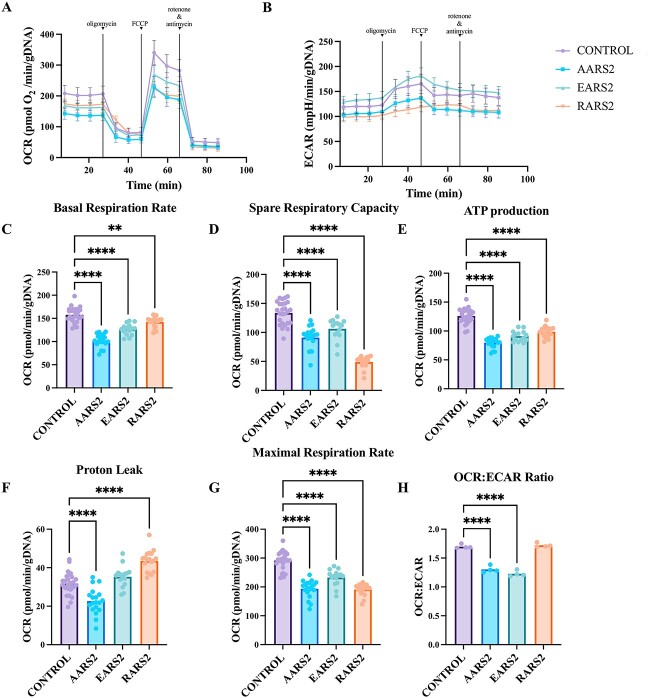
Mitochondrial respiration rate is altered in mutant mt-ARS iNPCs. Mitochondrial stress test result measured by Seahorse XFe96 analyser (A) oxygen consumption rate (OCR) in iNPCs carrying mt-ARS defects. (B) Extracellular acidification rate (ECAR). (C–G) Parameters relating to mitochondrial respiration calculated from OCR data. (H) Ratio of OCR to ECAR. Data was normalized to total DNA content. Data shown as mean ± SEM, ^*^P ≤ 0.05, ^*^^*^P ≤ 0.01, ^*^^*^^*^P ≤ 0.001, ^*^^*^^*^^*^P ≤ 0.0001, one-way ANOVA (Bonferroni).

To further characterize the impact of mt-ARS mutations on mitochondrial respiration, we measured the oxygen consumption rate (OCR) and the extracellular acidification rate (ECAR) in mutant mt-ARS and control iNPCs using the Seahorse XFe96 analyser. OCR is an indicator of electron transport chain (ETC) activity, and thus respiration, while ECAR correlates with the rate of glycolysis and lactate production [20]. Mutant *AARS2, EARS2* and *RARS2* iNPCs exhibited impaired mitochondrial respiration to various degrees (Fig. 3). *AARS2* and *EARS2* deficiency resulted in decreased basal and maximal respiration rates accompanied by a reduction in ATP production and lower spare respiratory capacity compared to controls. Reduction in OCR upon oligomycin-mediated inhibition of ATP synthase indicates that ATP production in these cells is primarily generated through mechanisms other than OXPHOS. Decreased OCR/ECAR ratios in *AARS2* and *EARS2* iNPCs indicate a stronger reliance on glycolysis, and increased lactate production. Similar metabolic defect may lead to increased lactate levels in the brain and CSF of patients [21–23]. In contrast, *RARS2* defect strongly reduced the spare respiratory capacity and maximal respiration, and increased proton leak, without affecting the OCR/ECAR ratio. This suggests that *RARS2* mutant iNPCs rely more on OXPHOS to generate ATP without readily switching to glycolysis upon ETC dysfunction and this compensation results in increased proton leak and poor OXPHOS coupling.

**Figure 4:**
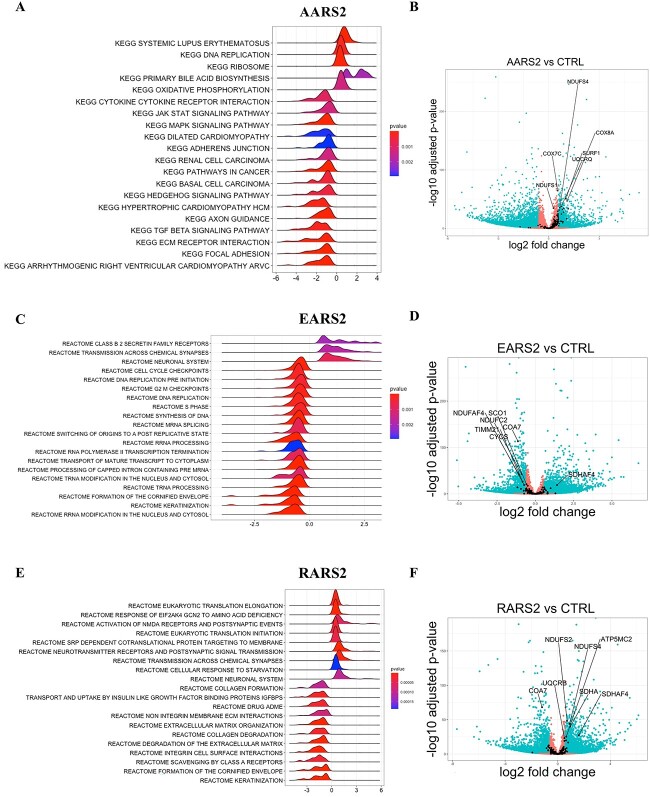
Pathway analysis of RNA sequencing data from MT-ARS iNPCs. Gene set enrichment analysis and volcano plot highlighting nuclear-encoded OXPHOS subunits, which increased in AARS2 (A and B), and RARS2 (E and F), but reduced in EARS2 (C and D) iNPCs.

**Figure 5:**
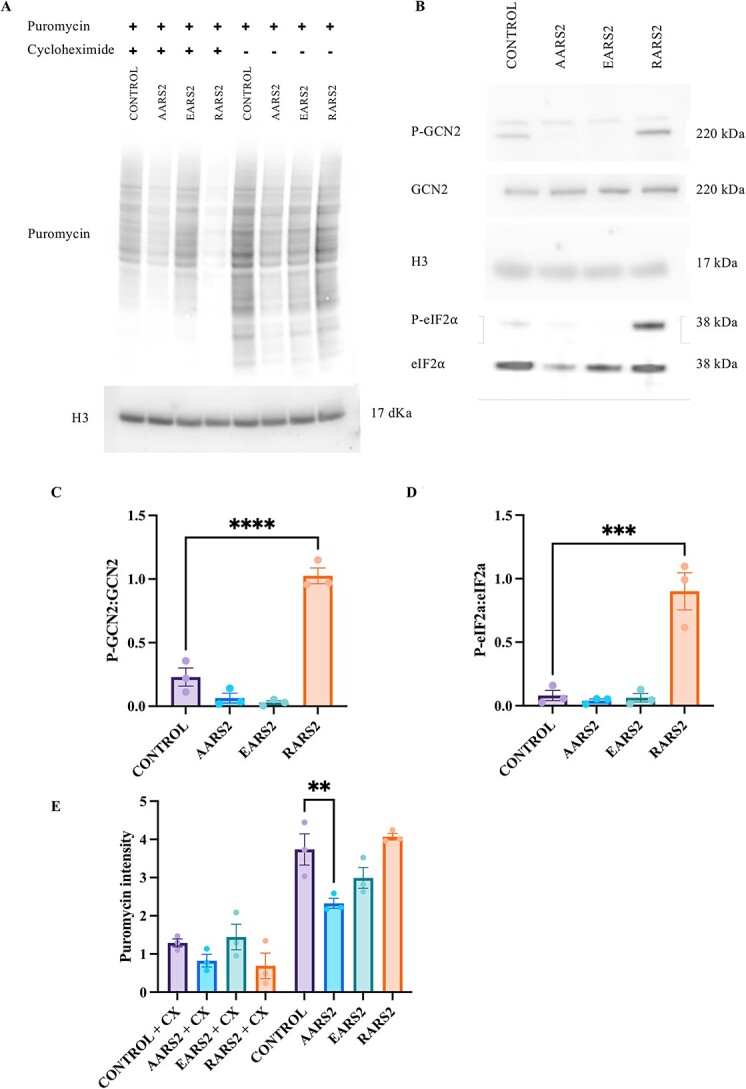
Mt-ARS defect alter protein translation rate and activate ISR in iNPCs. (A) Western blot image of the relative amount of puromycin incorporated into newly synthesized polypeptides during 10-min incubation of iNPCs. Cycloheximide (CX) was used as indicated as a negative control to block translation. H3 was used as a loading control. (B–D) Western blot and quantification of phosphorylated and total GCN2 and eIF2a in iNPCs. Activation of GCN2 and eIF2a shown as ratios of phosphorylated to total levels normalized to H3 as a loading control. (E) Quantification of puromycin blot. Puromycin signal was measured for each lane and normalized to H3 signal. Data shown as mean ± SEM of 3 independent experiments, ^*^P ≤ 0.05, ^*^^*^P ≤ 0.01, ^*^^*^^*^P ≤ 0.001, ^*^^*^^*^^*^P ≤ 0.0001, one-way ANOVA (Bonferroni).

**Figure 6:**
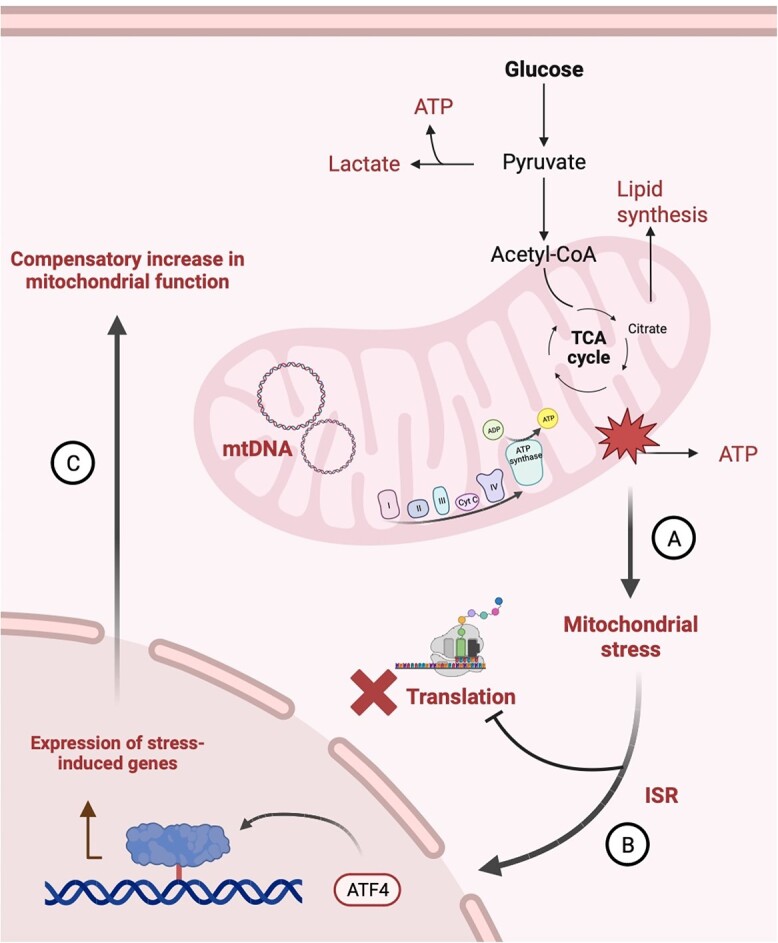
Summary of the proposed processes operating in iNPCs carrying mutations in mt-ARS. (A) Mt-ARS defects impair mitochondrial translation causing mitochondrial dysfunction which may include insufficient ATP production and activation of mitochondrial stress. (B) The integrated stress response may mediate the mitochondrial stress signal to the nucleus activating the expression of stress-induced genes to reach adaptive homeostasis and improve mitochondrial function while also inhibiting cytosolic protein translation. (C) Compensatory mechanisms aimed to improve mitochondrial function may include increasing mtDNA copy numbers or production of OXPHOS components. Affected cells may also shift their metabolism to glycolysis to meet ATP demand (as in AARS2 and EARS2 lines) causing a build-up of lactic acid.

### Transcriptomic analysis in mt-ARS mutant iNPCs detected impaired mitochondrial translation, altered neuronal differentiation and ISR activation

To further understand nuclear-mitochondrial crosstalk in mt-ARS mutant neurons, we obtained transcriptomic profiles by RNA sequencing (RNAseq) from the three iNPC lines carrying mt-ARS mutations and three healthy controls in triplicates per sample. Our results highlight three distinct transcriptomic profiles suggesting that defects in *AARS2, EARS2* and *RARS2* affect different biological processes and signalling pathways. Some pathways were shared across multiple databases including REACTOME and KEGG (see supplementary data).

Gene set enrichment analysis revealed altered biological processes and signalling pathways relevant to neurons and neuronal development in all three mt-ARS iNPC lines (Fig. 4). In *AARS2* iNPCs, we detected downregulation of genes involved in axon guidance, TGF-β and MAPK signalling, suggesting an impairment of early neuronal development and potentially affecting maturation of oligodendrocytes, which are in line with early-onset leukoencephalopathy seen in patients (Fig. 4A). Pathways related to neuronal development were upregulated in *EARS2* and *RARS2* mutant iNPCs compared to controls including axon guidance, neurogenesis, as well as synaptic development and neurotransmitter release (Fig. 4C and D). In addition, *RARS2* mutation activated pathways regulating synaptic plasticity, learning and memory, including FOXO signalling. *RARS2* iNPCs exhibit higher expression of *FOXG1* and lower expression of *SOX1* compared to other mutant lines and controls (Supplementary Fig. 11A). This may indicate that the *RARS2* mutant cells are more neuronal, while *AARS2* and *EARS2* mutations may impair neuronal differentiation. This is further supported by qPCR data showing that *AARS2* and *EARS2* mutant neurons exhibit lower expression of the neuronal marker NEUN compared to the control and *RARS2* mutant lines (Supplementary Fig. 12B), and lower expression of NESTIN in *AARS2* and *EARS2* iNPCs (Supplementary Fig. 12A).

Pathways such as mRNA processing, splicing, methylation, tRNA modifications, and rRNA processing were downregulated in *AARS2* and *EARS2* mutant lines compared to controls*,* which may be due to a defect of mitochondrial translation (Fig. 4A and C). An upregulation of cytoplasmic translation in *RARS2* mutant iNPCs is highlighted by increase in eukaryotic translation initiation and elongation, and decreased regulation of peptidase activity which facilitates translation by inhibiting the breakdown of new polypeptides (Fig. 4E). The upregulation of ISR targets such as GCN2 was most prominent in iNPCs with *RARS2* mutations. However, *AARS2* mutant iNPCs activated ISR by increased heme-regulated inhibitor (HRI) signalling (Supplementary Fig. 3A), likely triggered by protein aggregation [24, 25] and oxidative stress [26] leading to eIF2α phosphorylation and expression of downstream mediators to restore homeostasis. *AARS2, EARS2* and *RARS2* mutant lines all showed downregulation of cell cycle-related pathways.

### Mt-ARS defects affect signalling of the integrated stress response and cytosolic protein translation

The ISR is triggered by cellular stress (such as reduced mitochondrial translation) and usually decreases cytosolic protein synthesis, which enables some rescue of mitochondrial translation. We therefore investigated whether mt-ARS defects affected cytosolic protein translation. Cells were incubated with puromycin (10 μg/μl) for 10 min and compared to negative controls which were pre-treated with cycloheximide to block protein synthesis. Puromycin mimics the structure of tyrosine and thus becomes incorporated into nascent polypeptides [27]. Immunoblotting for puromycylated proteins therefore offers a convenient approach to visualize largescale disturbances in protein translation. We found that the rate of cytosolic protein translation was reduced by *AARS2* and *EARS2* mutations (Fig. 5A). However, cytosolic translation was unchanged in *RARS2* mutant iNPCs, which was accompanied by increased eIF2α and GCN2 phosphorylation (Fig. 5A–D).

## Discussion

Here we present systematic analyses of mitochondrial function in three different mt-ARS defects in *AARS2, EARS2* and *RARS2*, using a human in vitro model of neuronal cells, directly differentiated from human fibroblasts of patients with pathogenic mt-ARS mutations. Our results suggest that the three different mt-ARS defects share some molecular mechanisms of mitochondrial dysfunction, however different pathways observed in each of the three mt-ARS defects may highlight specific differences, in part explaining the clinical presentations.

A variable degree of respiratory chain defect has been detected in different tissues of patients with mt-ARS mutations. Patients with *AARS2* defects have been reported with a reduction in Complex IV activity [7] and reduced levels of Complex I, III and IV subunits in skeletal and cardiac muscle [23]. *EARS2* mutations lead to reduced enzymatic activity of Complex I, and IV in muscle and to a lesser extent in fibroblasts [22, 28]. In brain autopsy of a patient with *RARS2* variants a near-global cytochrome c oxidase-deficiency and severe deficiencies of complexes I, III, and IV have been reported [29]. Other patients with *RARS2* mutations had decreased Complex IV in fibroblasts [18, 30]. The respiratory chain defect was usually less severe in dividing cells (e.g. fibroblasts), while postmitotic tissues such as muscle, heart or brain presented with more severe abnormalities, suggesting that tissue specific differences of mitochondrial translation in post-mitotic tissues may contribute to the variability of the clinical symptoms. Previous studies have shown in several patients with biallelic mutations in mt-ARS genes that the mutations usually result in loss of function [3, 23, 30, 31]. In our study we observed more severe clinical presentation of the patients with biallelic missense mutations, suggesting that the molecular differences detected in our cellular models cannot be explained purely by the severity of the mutations.

In this study, all three iNPC lines showed altered OXPHOS on Seahorse assay, however, only *AARS2* mutations led to complex I, III and IV defects in postmitotic neurons, while *RARS2* and *EARS2* mutant neurons had normal respiratory chain subunits (Fig. 1). All three mutant iNPCs had abnormal transcriptional signatures, highlighting that both disease signatures and compensatory mechanisms enable the preservation of mitochondrial translation. Based on our data we suggest that proliferating iNPCs can compensate for mitochondrial translation defects to maintain balanced levels of OXPHOS components, which is more difficult in mature neurons.

What are the mechanisms which enable the preservation of mitochondrial translation in neurons with mt-ARS mutations? We detected increased mtDNA copy numbers in *AARS2* mutant iNPCs and *RARS2* mutant neurons (Fig. 2). Higher amounts of mtDNA could improve mitochondrial bioenergetics, but also lead to increased mt-tRNA levels, which may have a positive effect on mitochondrial function and translation. In support of our findings, increased mtDNA levels delayed disease onset in the mouse model carrying the mt-tRNA^Ala^ m.5024C>T mutation [32, 33].

Mitochondrial DNA (mtDNA) replication is regulated during neuronal differentiation to ensure that cells with a high requirement for ATP generated via OXPHOS have high mtDNA copy number, whereas those with a low requirement have fewer copies [34]. We showed that mtDNA copy number is increasing during neuronal differentiation in controls and *RARS2* mutant neurons, but not in *AARS2* and *EARS2* defect neurons (Fig. 2C). We think that it is most likely due to the delayed neuronal differentiation possibly caused by the mutations in these two cell lines supported by their RNAseq signatures [35].

Changes in mitochondrial respiration (on Seahorse) despite normal steady state levels of OXPHOS in mutant *AARS2, EARS2* and *RARS2* iNPCs further support that iNPCs are affected, but can compensate for the defect by cellular adaptations in all three mt-ARS defects. *AARS2* and *EARS2* defect iNPCs exhibited reduced rates of basal mitochondrial respiration compared to controls and appeared to be more glycolytic, indicating increased lactate production. It is in line with the clinical presentations of leukoencephalopathy with high lactate in several brain regions (Fig. 3). The hallmark of *EARS2* mutations is leukoencephalopathy with thalamus and brainstem involvement and high lactate (LTBL) [21, 22], and similar leukoencephalopathy with high lactate was also observed in patients with *AARS2* mutations. Both defects frequently result in increased CSF lactate levels [7]. Interestingly, *AARS2* and *EARS2* mutant iNPCs showed problems with neuronal differentiation, which may in part explain their different metabolic characteristics and the lack of compensatory mtDNA replication. In contrast, *RARS2* iNPCs appeared most vulnerable to pharmacological inhibition of the ETC indicating higher reliance on OXPHOS compared to *AARS2* and *EARS2* mutant neurons. These results suggest that *RARS2* mutant iNPCs compensate for their mitochondrial dysfunction to meet ATP demand possibly reflected by a greater proton leak and significantly reduced spare respiratory capacity compared to controls. It has been shown that proton leak measured by Seahorse regulates reactive oxygen species (ROS) [36], which can damage mitochondrial and cellular proteins, lipids, and nucleic acids [37].

*AARS2* and *EARS2* mutant iNPCs showed impaired neuronal development (e.g. MAPK and TGF-β), and downregulation of pathways that control gene expression (including RNA regulation, mRNA splicing, and tRNA processing) (Fig. 4). TGF-β and MAPK signalling regulate neuronal development and are vital for neuronal differentiation from NPCs and determine neural patterning [38, 39]. In the developing cortex, MAPK has been shown to regulate neurogenesis [40], and stimulate oligodendrocyte development [41]. The downregulation of TGF-β and MAPK pathways in our analysis indicates that the *AARS2* defect may directly impair early neuronal development and maturation of oligodendrocytes, which may be linked to early disease onset, neuronal specificity and white matter abnormalities seen in *AARS2* patients.

Recent studies implicate tRNA modifications as a new mechanism in synchronizing translation across the two compartments [42]. Furthermore, the integrated stress response (ISR) has recently emerged as a pathway activated in response to mitochondrial stress but precisely how mitochondria activate the ISR is still largely unknown [43, 44]. Upon ISR activation, distinct kinases [45, 46] converge on eIF2α to initiate specific transcription programmes orchestrated by ATF4 downstream to restore cellular function [47]. For example, mitochondrial dysfunction [48], as well as the accumulation of uncharged cytosolic tRNA molecules have been previously shown to activate general control nonderepressible 2 kinase (GCN2), an eIF2α kinase, to inhibit protein translation [49–52]. We therefore hypothesized that GCN2 signalling may be operating in mt-ARS patients. Gene set enrichment analysis revealed upregulation of two ISR branches—the HRI and GCN2 pathways in *AARS2* and *RARS2* mutant iNPCs respectively (Supplementary Figs 3A and 10B). We could detect increased GCN2 phosphorylation in *RARS2* but not in *AARS2* and *EARS2* mutants, suggesting that ISR activation can be triggered by different mechanisms in diverse mt-ARS defects (Fig. 5B–D).

Mitochondrial translation defects may be compensated by adjusting cytosolic translation to the needs of the cell, which may be regulated by the ISR or tRNA modifications [53]. Therefore, we studied cytosolic translation in mt-ARS mutant iNPCs. The relative amount of puromycylated proteins (i.e. proxy for the protein translation rate) was lower in *AARS2* and *EARS2* mutations, suggesting reduced cytosolic protein synthesis in order to compensate for the cellular dysfunction (Fig. 5A). However, no reduction, but different expression profile was observed in *RARS2* mutant iNPCs, suggesting different regulatory mechanisms, or different phases of cellular stress response. This effect is likely mediated by pathways identified through RNA sequencing showing upregulation of eukaryotic translation in *RARS2* defect iNPCs (Fig. 4E). Since the ISR is activated by various triggers depending on the type of stress, these triggers may be cell-type dependent and affected by differentiation. For example, mitochondrial stress has been shown to selectively activate the ISR via the HRI branch in Schwann cells [54], and distinct mitochondrial defects trigger the ISR depending on the stage of differentiation and metabolic state of the cell (e.g. mitochondrial inner-membrane hyperpolarization vs GCN2 activation by ETC inhibition) [55]. Therefore, we must also consider these factors as potential reasons for the differences in ISR activation among our three mt-ARS models.

The reduction in protein translation rate detected in *AARS2* and *EARS2* mutant iNPCs could be possibly mediated by HRI [56, 57] shifting towards a more glycolytic state, decreased cytosolic protein translation, and reduced cell proliferation indicated by the downregulation of cell cycle related pathways. In neuronal progenitors this adaptation may be beneficial in the short term [58]. In the case of EARS2 and AARS2 we observed decreased protein synthesis rates despite no evidence of increased eIF2α phosphorylation. It is possible that these cells underwent a transitory stress which would explain why we see translation attenuation of certain targets which persist even after global translation recovery [59]. However, in RARS2 mutant cells, despite the activation of eIF2α we have not detected decreased cytosolic translation. Indeed, certain cellular conditions have been shown to enable the bypass of eIF2α-mediated translational inhibition, restoring protein translation during long term activation of eIF2α favouring translation of long-lived proteins [59]. Maintaining normal translation rates in RARS2 mutant cells may be the result of an adaptive stress response modulating the protein translation profile to attempt cellular rescue. Furthermore, studies have shown that a high-level ectopic expression of an eIF2α kinase are required to completely block translation and cell growth, while lower levels of eIF2α phosphorylated after mild stresses, can lead to no effect observed on global translation [60–62]. Taken together, HRI and GCN2 activation may represent two mechanisms that mediate adaptive effects on cellular homeostasis caused by distinct mitochondrial defects resulting from different mt-ARS variants (see Fig. 6).

In summary, we have successfully generated and characterized neuronal progenitor cells from patients carrying rare variants in three different mt-ARS genes affecting the nervous system. Our neuronal models allow the investigation of compensatory mechanisms underlying neurological phenotypes of patients with mt-ARS defects in a more physiological and tissue-specific manner than previously reported in fibroblasts [7, 18, 22]. Defects in *AARS2, EARS2* and *RARS2* trigger unique compensatory mechanisms in iNPCs which may involve specific branches of the ISR. Whether the activation of different compensatory mechanisms in mt-ARS are gene specific, or simply related to the severity of the mitochondrial translation defect in the different mt-ARS models need further investigations.

## Materials and methods

### Experimental model and cell culture

Induced neural progenitor cells (iNPCs) were generated by direct conversion of fibroblasts from 3 healthy controls and patients compound heterozygous for *AARS2* mutations (c.1188G>A; c.1709delG), *EARS2* mutations (c.322C>T; c.334G>C), or homozygous for a *RARS2* variant (c.392T>G) using the method published by Meyer *et al*. [63]. For direct conversion, collected fibroblasts were seeded for tissue culture and treated with Sendai virus (Cytotune kit, ThermoFisher) to express OCT3, SOX2, KLF4, and C-MYC for 12 h. The medium was switched to NPC medium containing FGF2 and EGF 48 h after transduction. Cells were cultured at 37°C with 5% CO_2_ in Matrigel-coated six-well plates and maintained using Accutase Cell Dissociation Reagent (ThermoFisher) and STEMdiff Neural Progenitor Medium supplemented with 10 μM Y-27632 (AdooQ BioScience) to increase the survival of iNPCs by blocking apoptosis after passaging. The resulting iNPCs were differentiated into a mixed population of excitatory and inhibitory forebrain-type neurons for selected experiments as indicated using commercially available STEMdiff Forebrain Neuron Differentiation and Maturation Kit (STEMCELL Technologies). Neuronal differentiation has been fully successful only in one of the three control iNPCs, therefore we were only able to validate the experiments in neurons using one control line, which is a limitation of the data on neurons. Neurons were differentiated for 15 days before harvesting. Cell cultures were monitored for mycoplasma using EZ-PCR Mycoplasma Test Kit (Geneflow) following manufacturer’s instructions.

### Western blotting for mitochondrial proteins

Whole cells were lysed in buffer containing 1% SDS, 50 mM Tris– HCl (pH 7.8), 150 mM NaCl, and Complete Mini-Roche protease inhibitors. BCA Protein assay (Thermo Fisher) was used to determine and equalize total protein concentration across samples. Heat-denatured samples were loaded onto precast NuPAGE 4%–12% Bis-Tris gels (Thermo Fisher) at 15 μg of protein per well. Loaded gels were run for 1–2 h at 150 V to allow for size-dependent protein separation. Proteins were then transferred to a PVDF membrane and blocked in a 5% milk solution. Membranes were incubated with primary antibodies overnight at 4°C. Primary antibodies (OXPHOS Human WB Antibody Cocktail; Anti-TOMM20; Abcam) were diluted in 5% milk in PBS supplemented with 0.1% TWEEN20. Membranes were subsequently incubated with HRP-coupled secondary antibodies (Goat anti Rabbit IgG; Invitrogen; Goat Anti-Mouse IgG; Abcam). Proteins were detected using Amersham ECL Detection Reagent (Life Technologies) and imaged using Alliance chemiluminescence imaging system (UVITEC). Quantification of relative signal intensities of OXPHOS complexes were normalized to TOM20 levels.

### Mitochondrial DNA copy numbers

DNA was extracted using Wizard Genomic DNA Purification Kit (Promega) following manufacturer’s instructions. Purified DNA was amplified using RT-qPCR and the relative amounts of mtDNA were calculated as mean ΔCt values of the difference in cycle threshold (Ct) of the mitochondrial encoded gene ND1 minus the Ct of the nuclear gene B2M.

### Assessing protein translation rate

The rate of global protein translation was quantified using a puromycin assay whereby puromycin is incorporated into nascent polypeptides and visualized with western blotting (Anti-Puromycin Antibody; EMD Millipore) [27, 64]. Control and mutant iNPCs were treated with puromycin (10 μg/ml) for 10 min. Negative controls were pre-treated with cycloheximide (50 μg/ml), a potent inhibitor of protein translation.

### RNA sequencing and pathway analysis

Patient derived iNPCs harbouring *AARS2*, *EARS2* or *RARS2* mutation and three healthy control iNPCs were used for RNA sequencing. Total RNA extraction for sequencing was carried out using the mirVanaTM miRNA isolation kit following manufacturer’s instructions. RNA-seq libraries were prepared with Takara’s SMARTer Stranded Total RNA-Seq Kit and RNA was sequenced with Novaseq platform with > 30 mio reads per sample. Sequencing reads were checked with FastQC for quality control. Raw data was trimmed with Trim Galore (v0.6.5) to remove adapter and low QC reads [65]. STAR (v2.7.3a) was used for splice-aware alignment and mapping to genome [66]. We applied FeatureCounts to quantify reads assigned to genes [67]. DESeq2 was then used to identify differentially expressed genes [68]. ClusterProfiler was used for GO and gene set enrichment analysis (GSEA) analysis [69]. Raw data available upon request.

### Mitochondrial respiration

We measured the oxygen consumption rate (OCR) using the Seahorse XFe96 Analyzer following manufacturer’s instructions. Control and mutant iNPCs were seeded in a Seahorse 96-well plate (Agilent Technologies) at a density of 4 × 10^5^ cells per well (minimum 20 wells per group). Cultures were switched to Seahorse XF DMEM Medium pH 7.4, supplemented with 5 mM Glucose, 1 mM sodium pyruvate, 2 mM L-Glutamine. Cells were maintained at 37C in a CO_2_-free incubator for one hour prior to OCR measurement. Oligomycin, FCCP, rotenone and antimycin A (Sigma) dissolved in DMSO were injected to each well to reach a final concentration of 1 μM. Data was normalized to total DNA (Hoechst 33342, 2 μM) and analysed using WAVE software (Agilent Technologies).

## Supplementary Material

supplementary_figures_pdf_ddad196
