# Supplementary material for: Mitochondrial aminoacyl-tRNA synthetases trigger unique compensatory mechanisms in neurons
Source: Hum Mol Genet. 2023 Nov 17;33(5):435–47. doi: 10.1093/hmg/ddad196 (PMC10877469; doi:10.1093/hmg/ddad196)
Supplement: supplementary_figures_pdf_ddad196 [file supplementary_figures_pdf_ddad196.pdf]

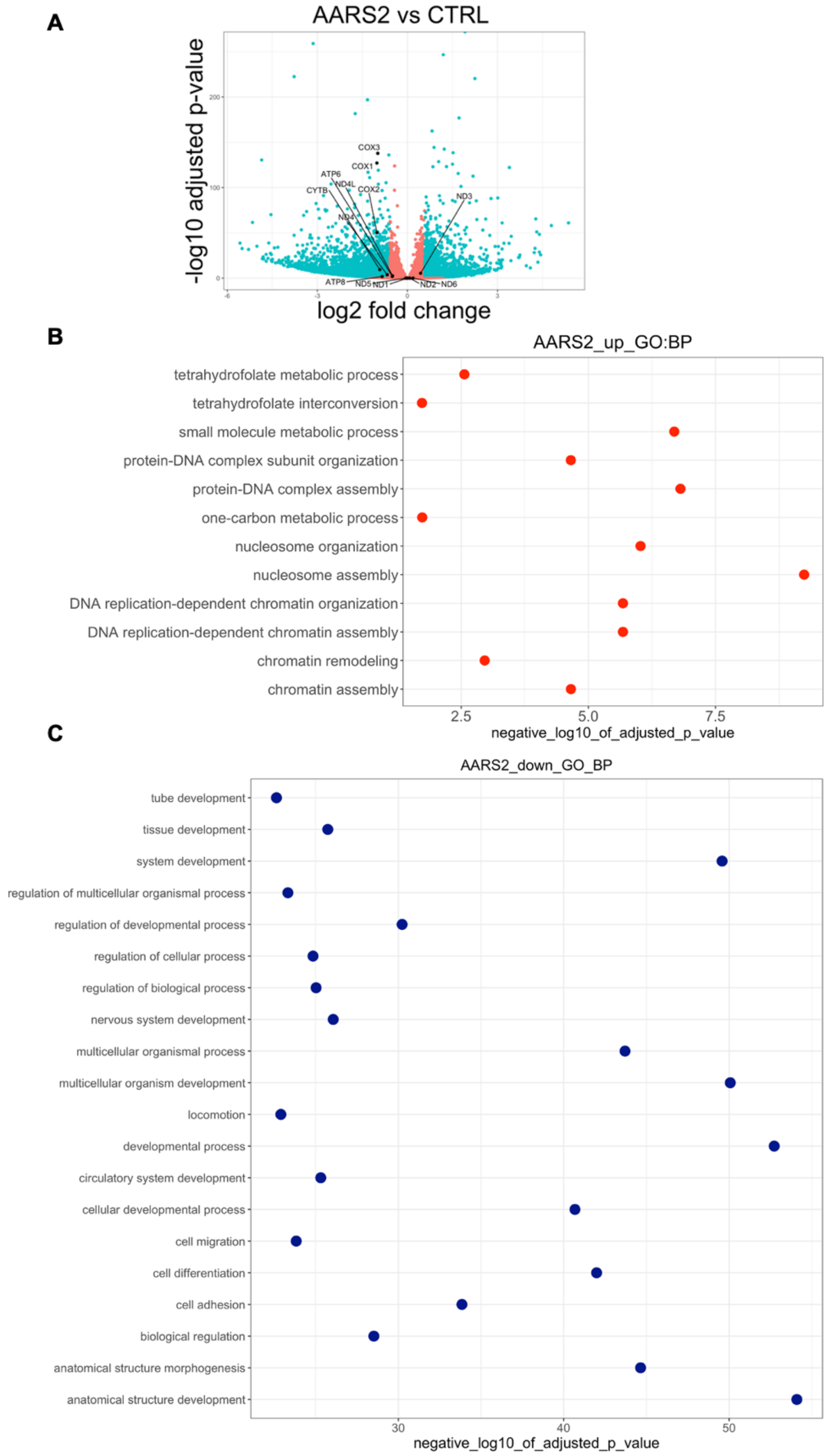

Supplementary Figure 1. Additional figures from AARS2 iNPC transcriptomic analysis.

(A) Volcano plot showing fold change in gene expression annotated with mtDNA-encoded transcripts. (B) Enriched GO terms obtained from upregulated transcripts. (C) Enriched GO terms obtained from downregulated transcripts.

A

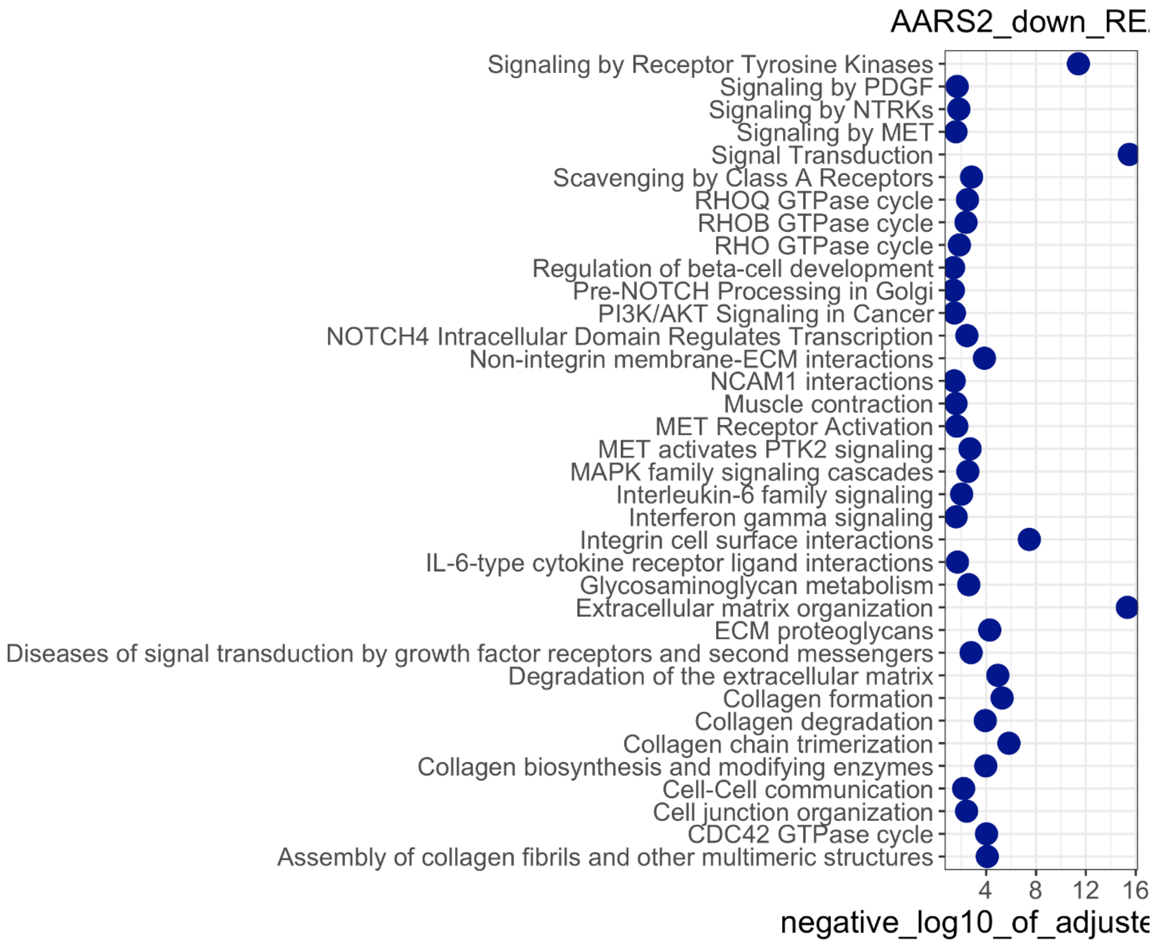

Supplementary Figure 2. Additional figures from AARS2 iNPC transcriptomic analysis.

(A) Pathway analysis results obtained from downregulated transcripts.

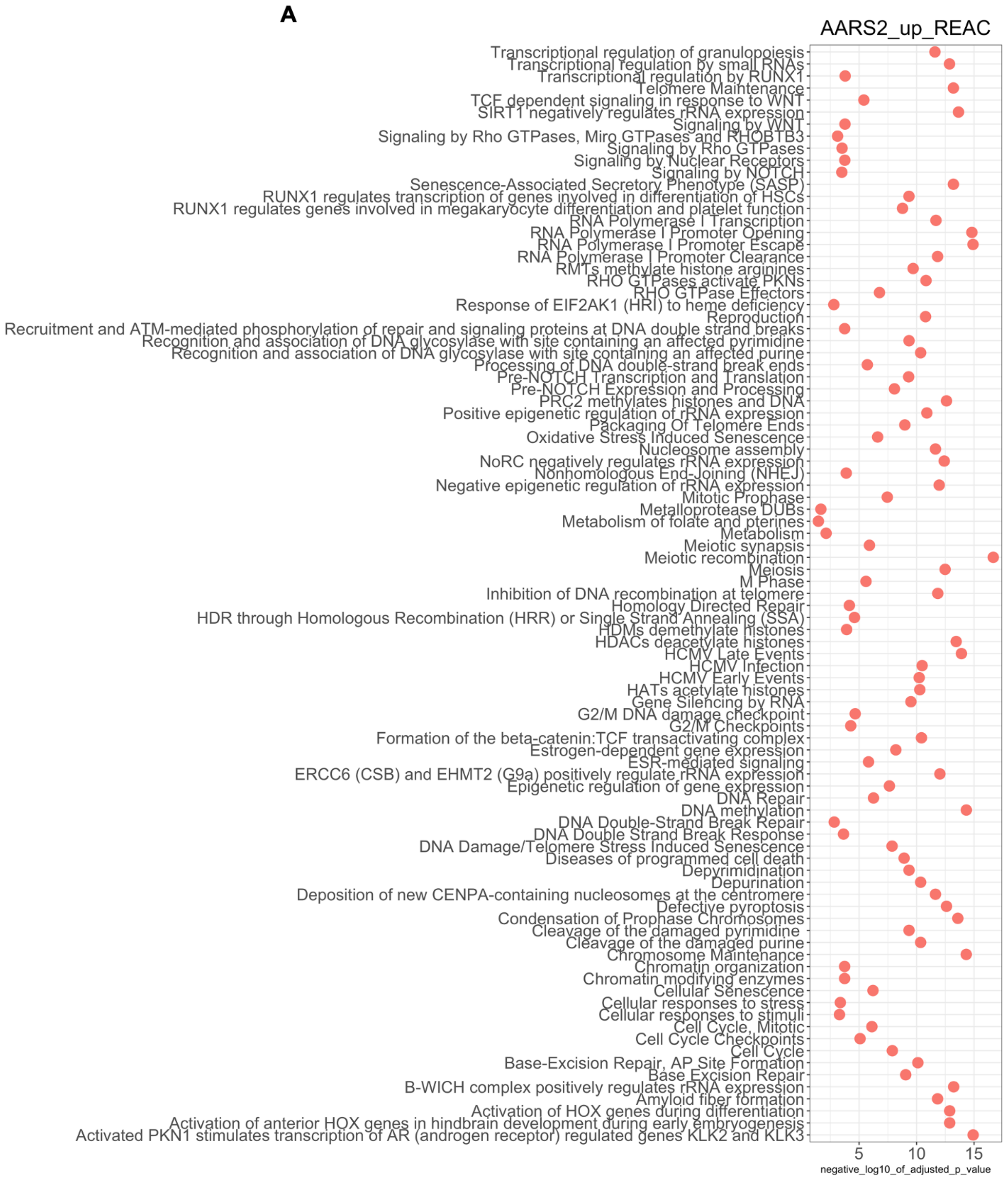

Supplementary Figure 3. Additional figures from AARS2 iNPC transcriptomic analysis.

(A) Pathway analysis results obtained from upregulated transcripts.

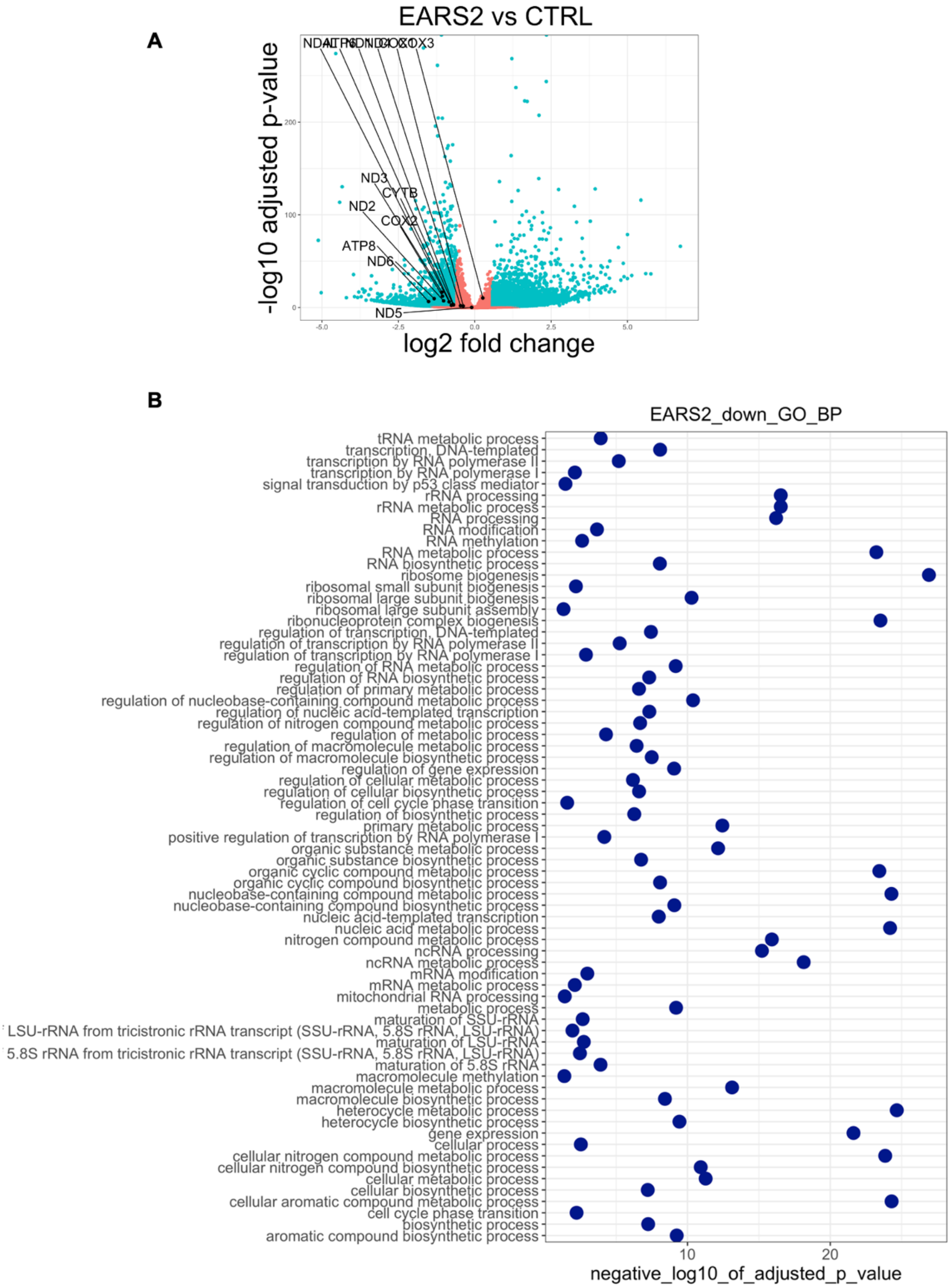

Supplementary Figure 4. Additional figures from EARS2 iNPC transcriptomic analysis.

(A) Volcano plot showing fold change in gene expression annotated with mtDNA-encoded transcripts. (B) Enriched GO terms obtained from downregulated transcripts.

A

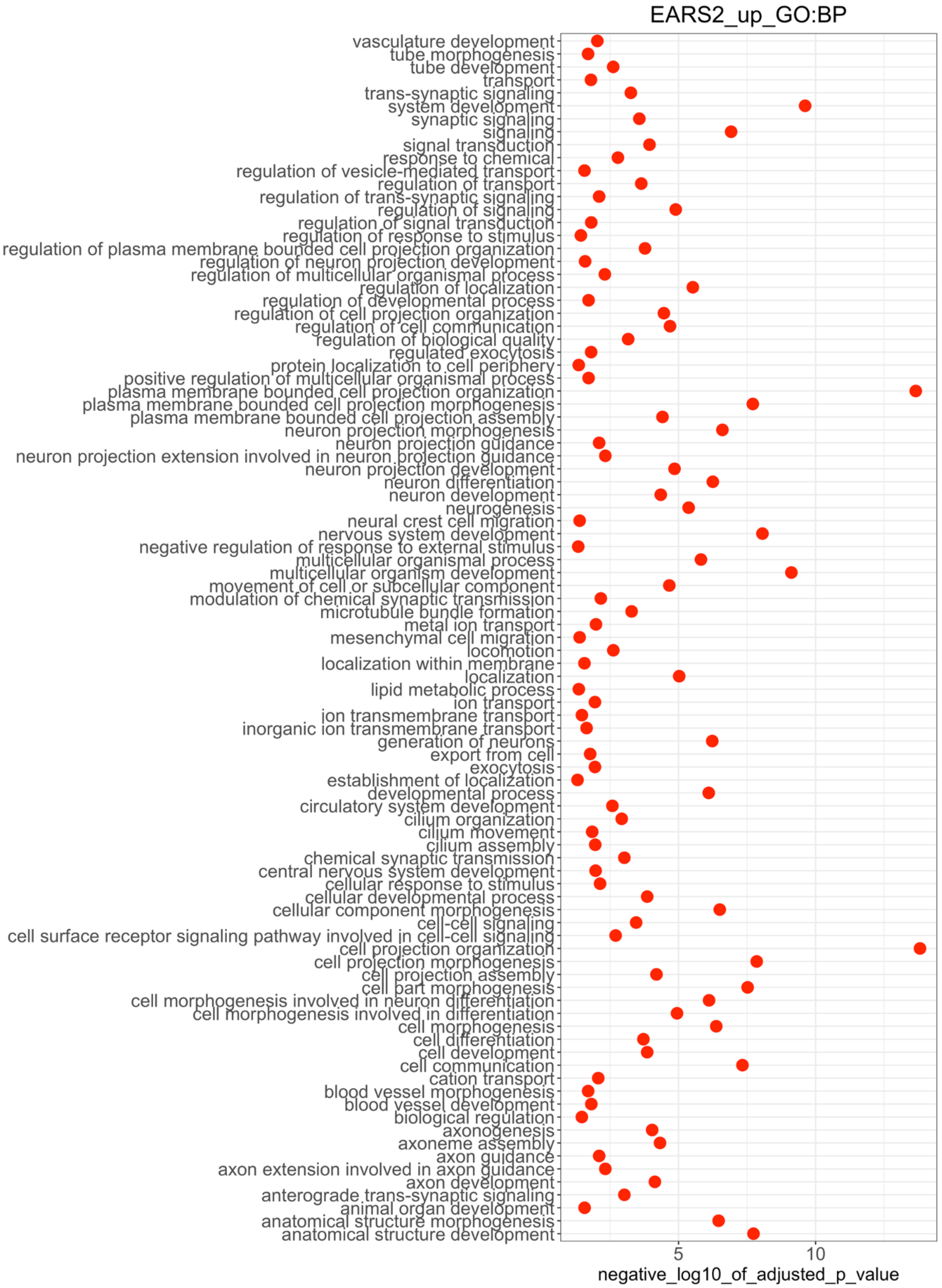

Supplementary Figure 5. Additional figures from EARS2 iNPC transcriptomic analysis.

(A) Enriched GO terms obtained from upregulated transcripts.

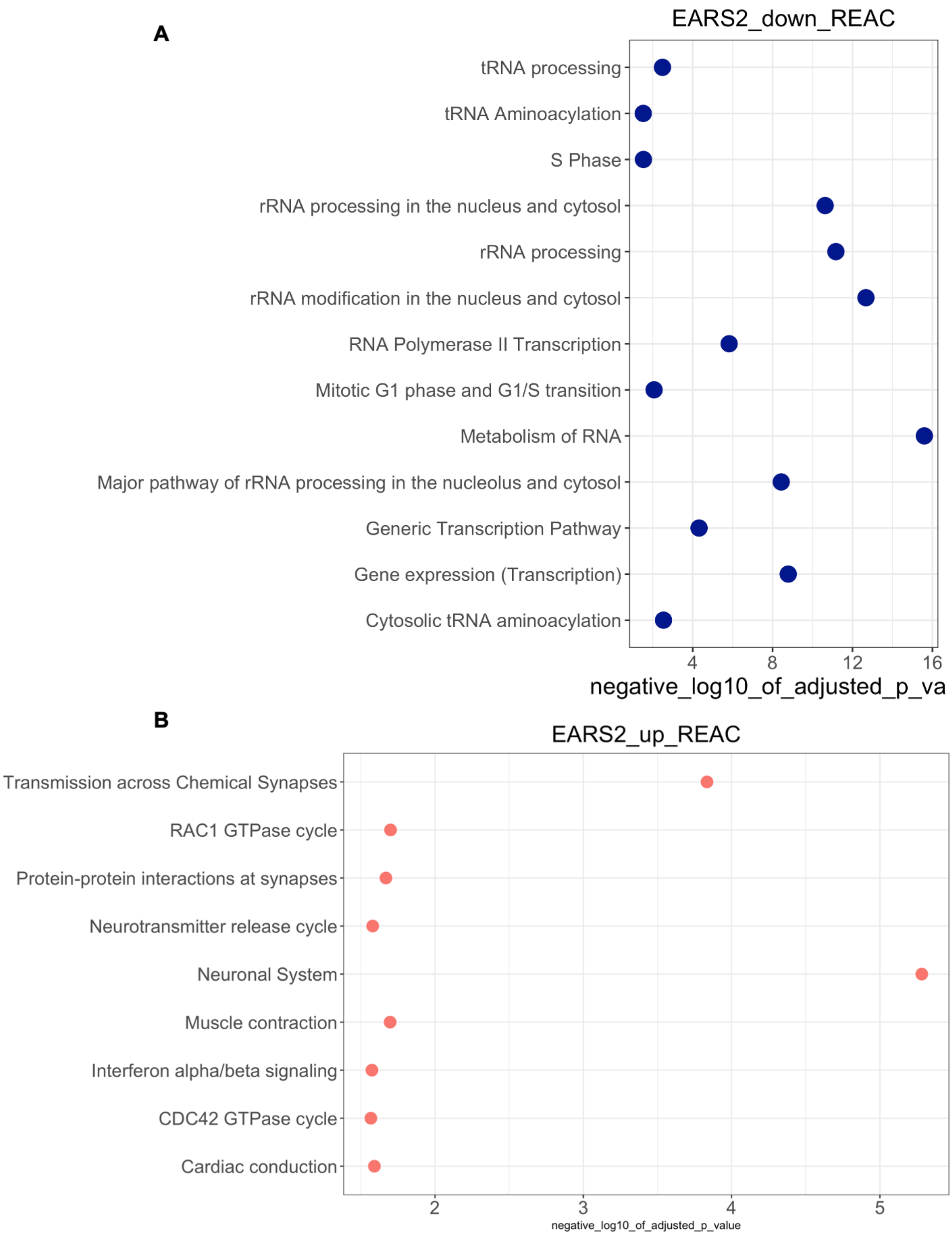

Supplementary Figure 6. Additional figures from EARS2 iNPC transcriptomic analysis.

(A) Pathway analysis results obtained from downregulated transcripts. (B) Pathway analysis results obtained from upregulated transcripts.

A

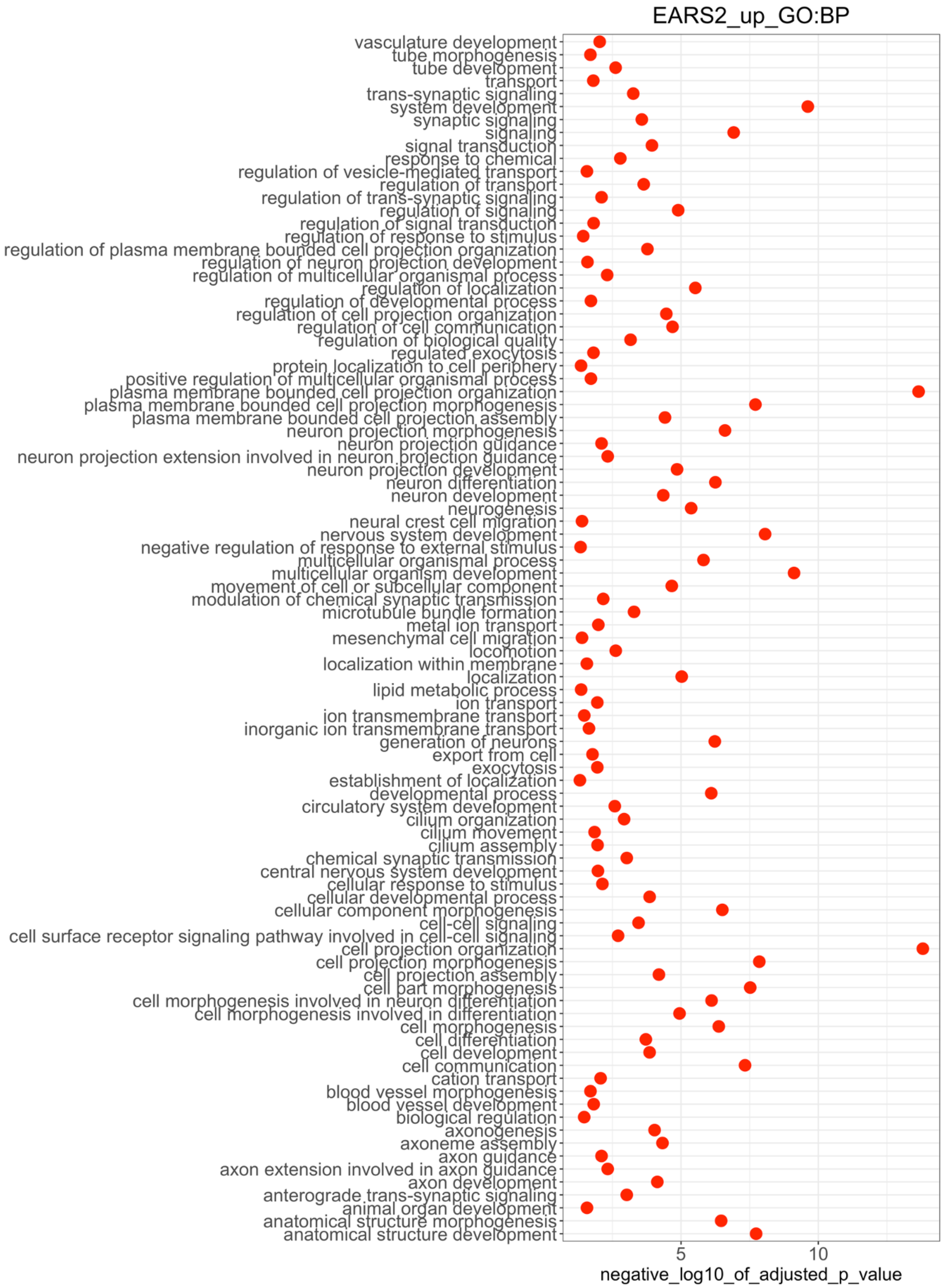

Supplementary Figure 7. Additional figures from EARS2 iNPC transcriptomic analysis.

(A) Pathway analysis results obtained from upregulated transcripts.

A

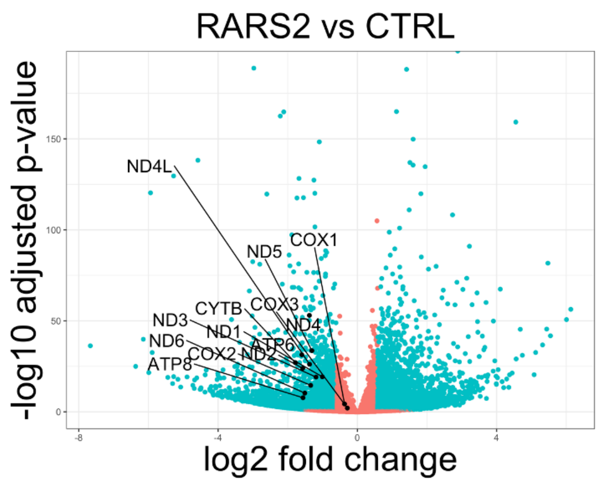

B

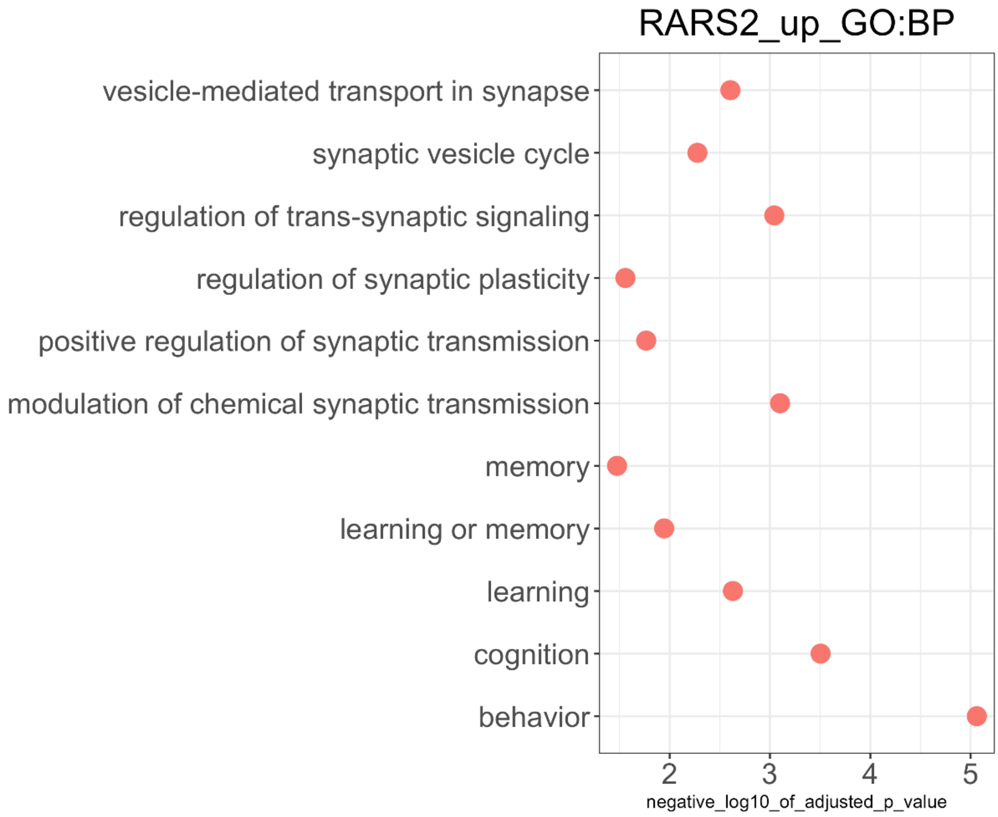

Supplementary Figure 8. Additional figures from EARS2 iNPC transcriptomic analysis.

(A) Volcano plot showing fold change in gene expression annotated with mtDNA-encoded transcripts. (B) Enriched GO terms obtained from upregulated transcripts.

A

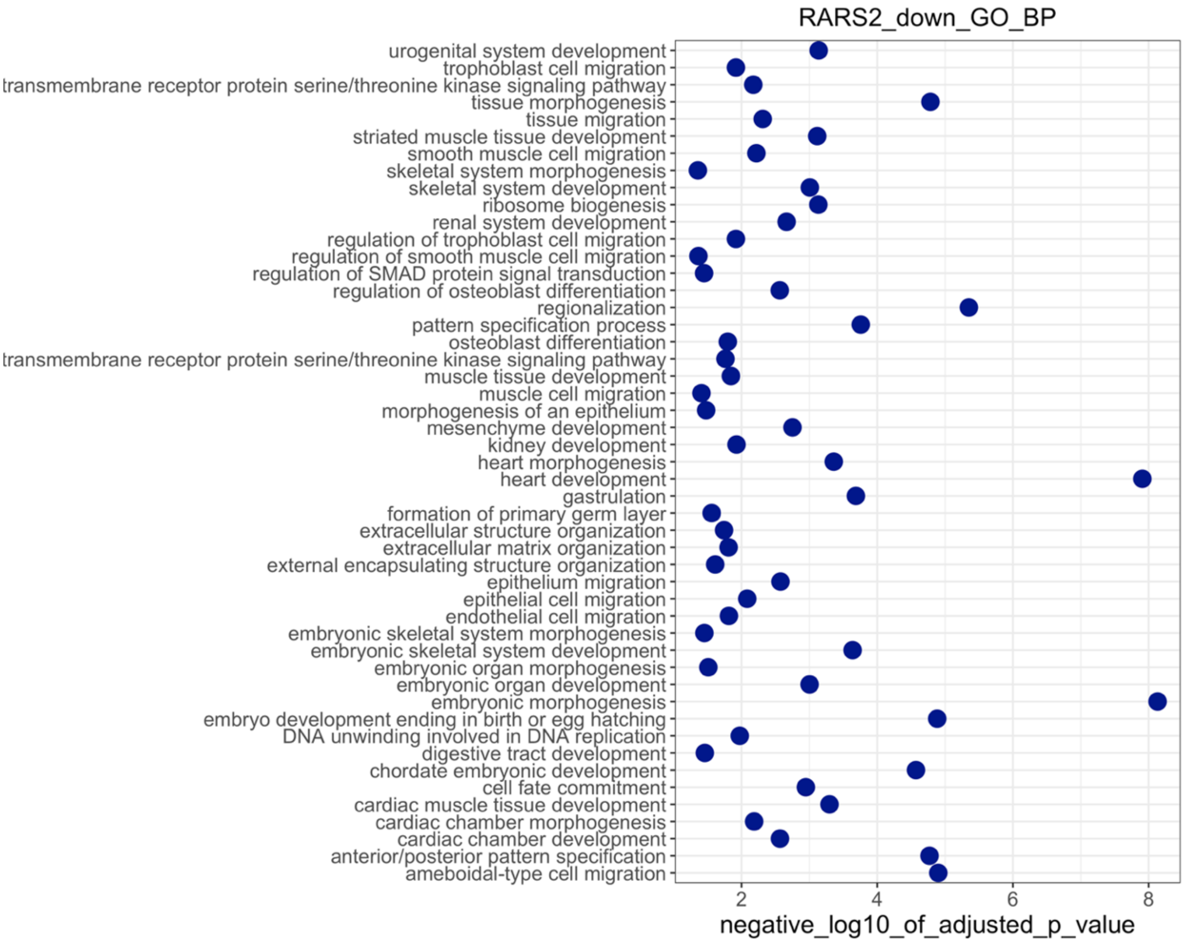

Supplementary Figure 9. Additional figures from RARS2 iNPC transcriptomic analysis.

(A) Enriched GO terms obtained from downregulated transcripts.

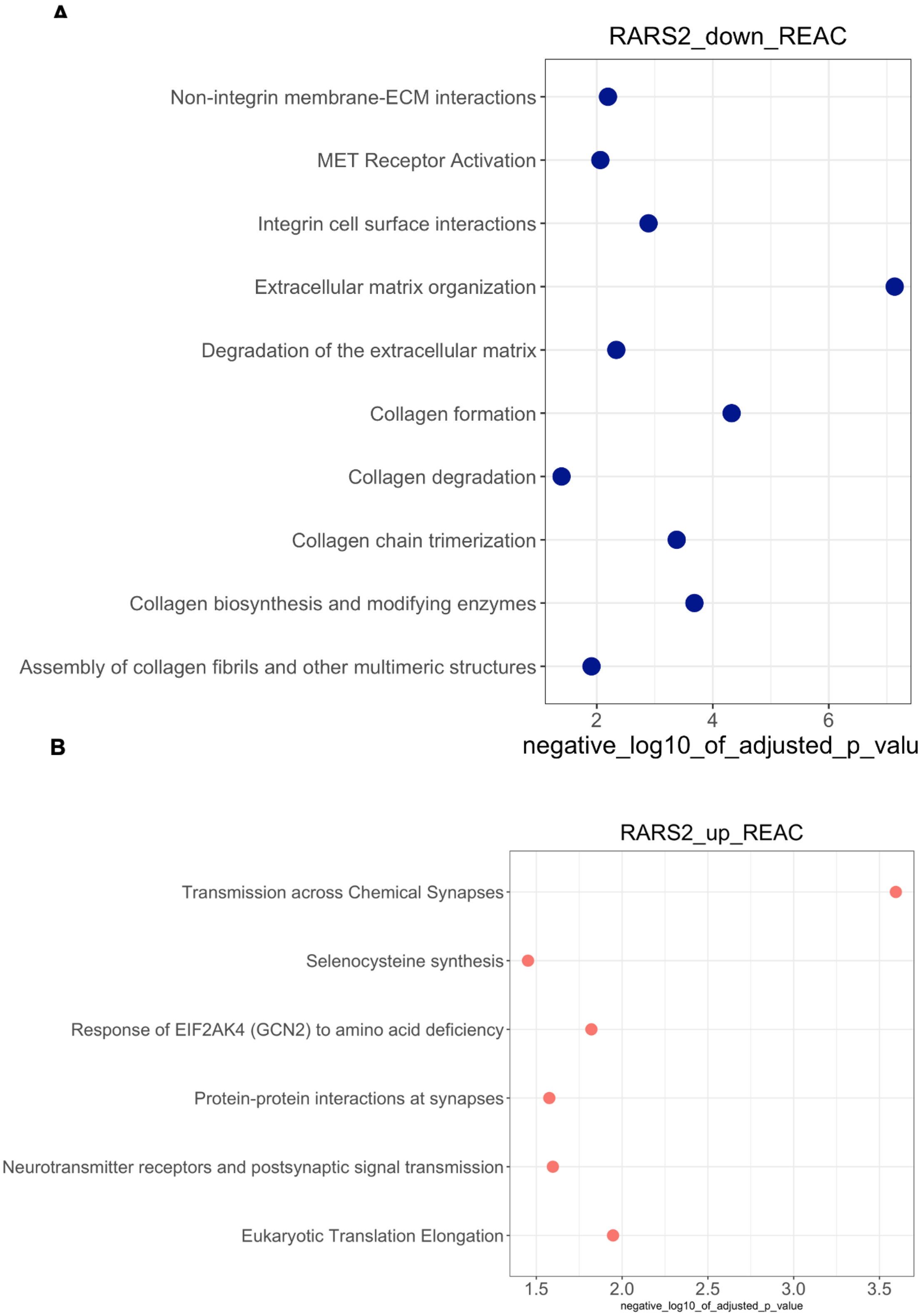

Supplementary Figure 10. Additional figures from RARS2 iNPC transcriptomic analysis.

(A) Pathway analysis results obtained from downregulated transcripts. (B) Pathway analysis results obtained from upregulated transcripts.

A

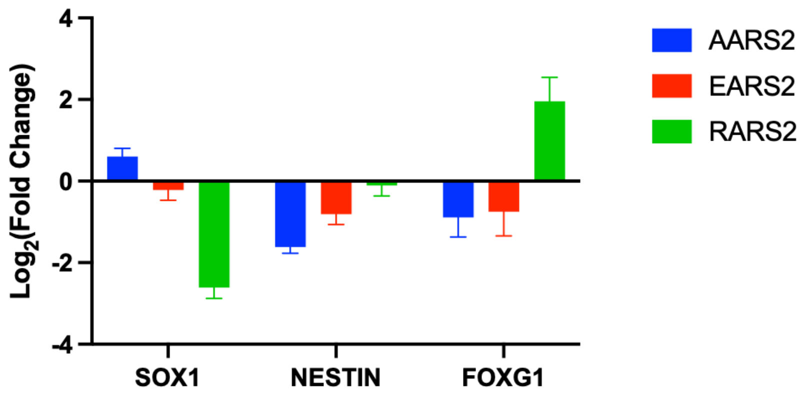

B

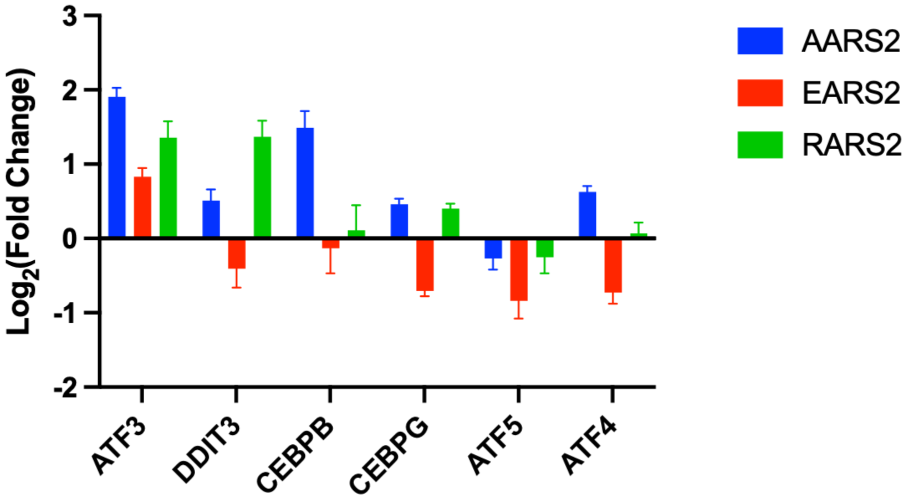

Supplementary Figure 11. Change in expression of selected relevant genes identified through RNA sequencing of MT-ARS iNPCs.

(A) Gene expression of markers for neuronal differentiation identified in MT-ARS iNPCs compared controls. (B) Gene expression of downstream integrated stress mediators of MT-ARS iNPCs compared controls.

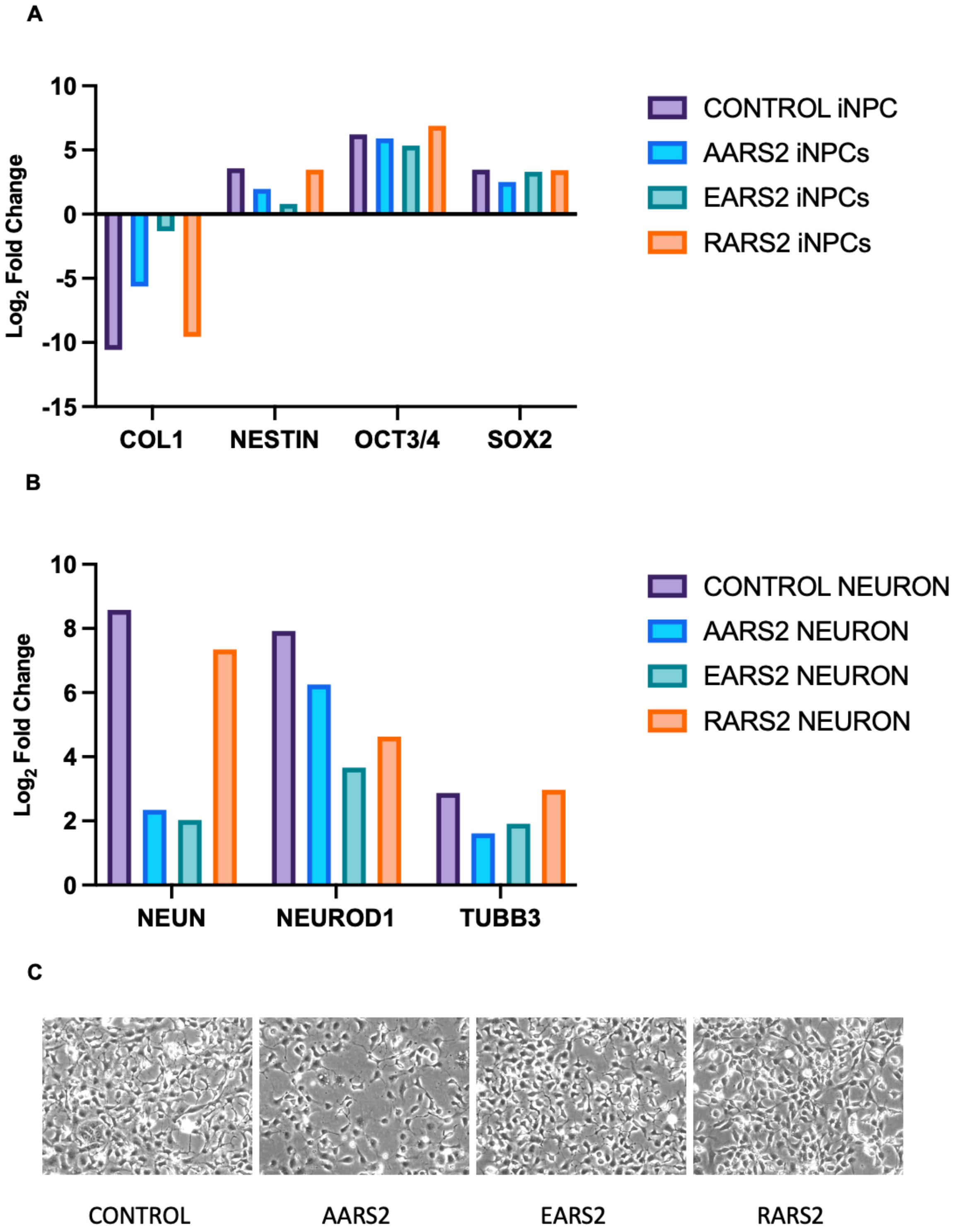

Supplementary Figure 12. Validation of neuronal differentiation

(A) Gene expression of markers for neuronal differentiation in iNPCs compared to fibroblasts measured by qPCR. (B) Gene expression of neuronal markers in neurons compared to iNPC levels measured by qPCR. (C) Images of iNPC cultures after neuronal induction showing typical morphology of proliferating neuronal progenitors.
